# Prognostic impact of circulating tumor cells assessed with the CellSearch System™ and AdnaTest Breast™ in metastatic breast cancer patients: the DETECT study

**DOI:** 10.1186/bcr3243

**Published:** 2012-08-15

**Authors:** Volkmar Müller, Sabine Riethdorf, Brigitte Rack, Wolfgang Janni, Peter A Fasching, Erich Solomayer, Bahriye Aktas, Sabine Kasimir-Bauer, Klaus Pantel, Tanja Fehm

**Affiliations:** 1 Department of Gynecology, University Medical Center Hamburg-Eppendorf, Martnistrasse 52, 20246 Hamburg, Germany; 2 Department of Tumor Biology, University Medical Center Hamburg-Eppendorf, Martnistrasse 52, 20246 Hamburg, Germany; 3 Department of Obstetrics and Gynecology, LMU, Maistrasse 11, 80337 Munich, Germany; 4 Department of Obstetrics and Gynecology, University Medical Center, Moorenstraße 5, 40225 Düsseldorf, Germany; 5 University Breast Center Franconia, Department of Gynecology and Obstetrics, Friedrich-Alexander University Erlangen-Nuremberg, Comprehensive Cancer Center Erlangen-Nuremberg, Universitätsstraße 21, 91054 Erlangen, Germany; 6 Department of Obstetrics and Gynecology, University Medical Center, Kirrberger Strasse 1, 66421 Homburg/Saar, Germany; 7 Department of Obstetrics and Gynecology, University Medical Center, Hufelandstrasse 55, 45147 Essen, Germany; 8 Department of Obstetrics and Gynecology, University Medical Center, Calwer Strasse 7, 72076 Tübingen, Germany

## Abstract

**Introduction:**

There is a multitude of assays for the detection of circulating tumor cells (CTCs) but a very limited number of studies comparing the clinical relevance of results obtained with different test methods. The DETECT trial for metastatic breast cancer patients was designed to directly compare the prognostic impact of two commercially available CTC assays that are prominent representatives of immunocytochemical and RT-PCR based technologies.

**Methods:**

In total, 254 metastatic breast cancer patients were enrolled in this prospective multicenter trial. CTCs were assessed using both the AdnaTest Breast Cancer and the CellSearch system according to the manufacturers' instructions.

**Results:**

With the CellSearch system, 116 of 221 (50%) evaluable patients were CTC-positive based on a cut-off level at 5 or more CTCs. The median overall survival (OS) was 18.1 months in CTC-positive patients. (95%-CI: 15.1-22.1 months) compared to 27 months in CTC-negative patients (23.5-30.7 months; p<0.001). This prognostic impact for OS was also significant in the subgroups of patients with triple negative, HER2-positive and hormone receptor-positive/HER2-negative primary tumors. The progression free survival (PFS) was not correlated with CTC status in our cohort receiving different types and lines of systemic treatment (p = 0.197). In multivariate analysis, the presence of CTCs was an independent predictor for OS (HR: 2.7, 95%-CI: 1.6-4.2). When the AdnaTest Breast was performed, 88 of 221 (40%) patients were CTC-positive. CTC-positivity assessed by the AdnaTest Breast had no association with PFS or OS.

**Conclusions:**

The prognostic relevance of CTC detection in metastatic breast cancer patients depends on the test method. The present results indicate that the CellSearch system is superior to the AdnaTest Breast Cancer in predicting clinical outcome in advanced breast cancer.

**Trial registration:**

Current Controlled Trials Registry number ISRCTN59722891.

## Introduction

Hematogenous tumor cell dissemination is a crucial step in tumor progression, and blood-derived metastases account for the majority of breast cancer-related deaths. Circulating tumor cells (CTCs) can be detected in the circulation. The detection of CTCs in blood can provide prognostic information [1,2]. Moreover, CTC detection and characterization have already improved our understanding of the complex process underlying tumor cell dissemination and metastatic progression in breast cancer. Cells that are able to disseminate into the circulation are of biologic relevance as potential founder cells for new metastases. The clinical potential of CTC detection is to stratify patients for therapies and to monitor treatment response with easy-to-perform blood tests. The first clinical studies that evaluate the clinical use of CTC detection are on the way. Different methods for the detection of CTCs have been described, but the optimal method for CTC detection is unclear so far [3,4].

At present, the CellSearch™ system (Veridex, LLC, Raritan, NJ, USA), which combines automated enrichment and immunostaining, is the only standardized technology to be approved by the US Food and Drug Administration (FDA) for the detection of CTCs in patients with metastatic breast, colon, or prostate cancer [2,5,6]. Another system that was developed is the AdnaTest BreastCancer™ (Adnagen AG, Langenhagen, Germany). This test is based on the detection of three tumor-associated transcripts (GA733-2, MUC-1, and HER2) by reverse transcription-polymerase chain reaction (RT-PCR) after immunomagnetic enrichment of tumor cells [7,8]. Both assays have been used in studies evaluating CTC detection [7-11]. However, a comparison of their prognostic value has not been performed in a prospective multicenter cohort with clinical follow-up. Therefore, the aim of the DETECT trial for patients with metastatic breast cancer was to directly compare the prognostic impact of these two commercially available CTC assayss, which are prominent representatives of immunocytochemical and RT-PCR-based technologies.

## Materials and methods

### Patients

A total of 254 metastatic breast cancer patients from nine German university breast cancer centers (Düsseldorf, Erlangen, Essen, Freiburg, Hamburg, Heidelberg, Munich, Regensburg, and Tübingen) were enrolled in this prospective, open-label, non-randomized study. Inclusion criteria were the following: epithelial invasive carcinoma of the breast with distant metastatic disease (M1); age of at least 18 years; availability of primary tumor tissue results for estrogen receptor (ER), progesterone receptor (PR), and human epidermal growth factor receptor 2 (HER2); and first diagnosis of metastatic disease or disease progression (before the start of a new treatment regimen). Blood was drawn before the start of a new line of therapy. The number of patients was based on funding resources.

Usually, therapy response was evaluated every 8 to 12 weeks by computed tomography scan, which was not mandatory. All patients gave their informed consent for the use of their blood samples. A web-based databank was designed for data management and online documentation. During the use of this interface, clinical investigators were blinded to the results of CTC testing, and the investigators performing CTC testing were blinded to the clinical data of the patients and the results of the CTC tests from the other centers. The study was approved by the ethics review board of the University of Tübingen (# 2007/B01). The trial was registered in the Current Controlled Trials Registry (# ISRCTN59722891).

### Detection methods for circulating tumor cells

Detection of CTCs and assessment of HER2 status of CTCs were performed by using both AdnaTest BreastCancer™ and the CellSearch™ system in accordance with the instructions of the manufacturers without modifications [5,10,11]. Sample preparation and analysis by AdnaTest BreastCancer™ were performed by either of two centers: the Department of Gynecology and Obstetrics of Essen (SK-B) or Tübingen (TF). CTC analysis by the CellSearch™ system was performed by either of two centers: the Department of Tumor Biology of University Medical Center at Hamburg-Eppendorf (KP and SR) or the Department of Gynecology at Munich (BR). In a previous validation study, these centers demonstrated that samples could be stored and transported (up to 72 hours), and examined the high inter- and intra-assay concordance of the results in a multicenter setting [5].

Before the study was started, each breast cancer center was assigned to send its samples only to the designated laboratory for the CellSearch™ system and AdnaTest BreastCancer™, respectively. Blood samples for AdnaTest BreastCancer™ were shipped in cooled boxes at 4°C, whereas samples for the CellSearch™ system were sent at room temperature in accordance with the recommendation of the manufacturer. All blood samples were processed within 48 hours for AdnaTest BreastCancer™ and 96 hours for the CellSearch™ system or were otherwise discarded. Both AdnaTest BreastCancer™ and the CellSearch™ system were performed independently, and the investigators were blinded to the results obtained by the other method.

### Circulating tumor cell detection with the AdnaTest BreastCancer™

Ethylenediaminetetraacetic acid (EDTA) blood samples were collected for CTC isolation by using the AdnaCollect™ blood collection tubes (Adnagen AG) and stored at 4°C until further analysis. Establishment and validation of AdnaTest BreastCancer™ have been described in detail elsewhere [7,8,11]. In brief, blood samples were incubated with a ready-to-use antibody mixture (against GA 73.3 and MUC1) - commercialized as AdnaTest BreastCancer Select™ (Adnagen AG) - in accordance with the instructions of the manufacturer. The labeled cells were extracted by a magnetic particle concentrator. Subsequently, mRNA isolation from lysed enriched cells was performed with a Dynabeads mRNA Direct™ Micro Kit (Dynal Biotech GmbH, Hamburg, Germany). Sensiscript™ Reverse Transcriptase (Qiagen GmbH, Hilden, Germany) was used for the reverse transcription in combination with oligo(dT)-coupled Dynabeads of the mRNA Direct™ Micro Kit (Dynal Biotech GmbH) [11]. The analysis of tumor-associated mRNA isolated from CTC tumor cells was performed in a multiplex polymerase chain reaction (PCR) for three tumor-associated transcripts (HER2, MUC1, and GA733-2) and the housekeeping gene β-actin. GA 73.3 refers to the epithelial cell adhesion molecule (EpCAM) epitope, and the GA733-2 transcript refers to EpCAM mRNA. The primers generate fragments of the following sizes: GA733-2, 395 base pairs (bp); MUC1, 293 bp; and HER2 and actin, 114 bp. Visualization of the PCR fragments was carried out with a 2100 Bioanalyzer (Agilent Technologies Inc., Santa Clara, CA, USA) by using DNA 1000 LabChips and the Expert Software Package (version B.02.03.SI307). The test was considered CTC-positive if a PCR fragment of at least one tumor-associated transcript (MUC-1, GA 773-2, or HER2) and a fragment of the control gene β-actin (internal PCR control) were clearly detected (peak concentration of greater than 0.15 ng/µL) in both blood samples. CTCs were considered HER2-positive if a PCR fragment of the HER2 transcript (peak concentration of greater than 15 ng/µL) was present. Twenty-five blood samples had to be excluded because of insufficient blood volume (n = 12), failure of the assay to pass quality control (n = 7), or time until processing of more than 48 hours (n = 6).

### Circulating tumor cell detection with the CellSearch™ system

Blood samples were collected into CellSave tubes (Veridex, LLC). The CellSearch Epithelial Cell Test (Veridex, LLC) was applied for CTC enrichment and enumeration. The method has been described in detail elsewhere [5]. In brief, CTCs are captured from peripheral blood by anti-EpCAM-antibody-bearing ferrofluid and subsequently identified by cytokeratin positivity/negativity for the leukocyte common antigen CD45 and 4',6-diamidino-2-phenylindole (DAPI) staining to ensure the integrity of the nucleus. A blood sample was positive when at least 5 CTCs - the prognostically relevant cutoff as previously published [1,9] - were present. HER2 expression of CTCs was characterized within the CellSearch™ system by the addition of a fluorescein-labeled anti-HER2 antibody (CellSearch™ tumor phenotyping reagent HER2; Veridex, LLC), as described previously [10,12,13]. Nine samples had to be excluded for technical issues: test failure (n = 6), hemolytic blood samples (n = 2), and insufficient blood volume (n = 1).

### Statistical analysis

The primary endpoint of the study was the rate of HER2-positive CTCs with each method that was reported shortly after the end of patient recruitment [14]. Secondary endpoints were the concordance between the two methods in HER2-positive CTC detection and the correlation of CTC detection with clinical follow-up: overall survival (OS) and progression-free survival (PFS). The study was performed in accordance with criteria of REMARK (**reporting recommendations **for **tumor marker prognostic studies**) [15,16]. Fisher's exact test was used to evaluate the relationship, and *P *values of less than 0.05 indicated statistical significance. The PFS and OS were estimated by using the Kaplan-Meier method. The log-rank test was used to compare PFS and OS between groups. A Cox proportional hazards regression model was used to predict death. Variables considered for the multivariate model, selected as significant in the univariate model, were CTC count, number of metastatic sites, molecular subtype, and metastatic site. The final model was selected by backward-stepwise regression. Hazard ratios for the Cox model and odds ratios for the logistic model were calculated with their 95% confidence intervals (CIs). A *P *value of less than 0.05 was considered statistically significant. Statistical analysis was performed by using SPSS version 20 (SPSS Inc., Chicago, IL, USA).

## Results

### Patient characteristics

The clinical characteristics of the patients and positivity rates for CTCs were described previously [14]. In brief, 69% of the patients had ER-positive and 35% had HER2-positive primary tumors; 38% received first-line, 26% second-line, and 36% third- or further-line treatment for metastatic disease. From June 2007 to September 2009, 267 patients with a median age of 57 years were included.

### Prognostic impact of the CellSearch™ system

**Figure 1:**
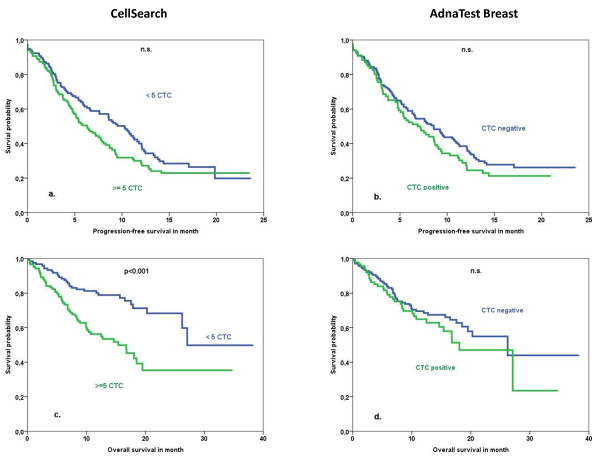
**Patient outcome with the two different tests for CTC detestion**. Progression-free survival for **(a) **CellSearch™ and **(b) **AdnaTest BreastCancer™. Overall survival for **(c) **CellSearch™ and **(d) **AdnaTest BreastCancer™. CTC, circulating tumor cell; n.s., not significant.

**Figure 2:**
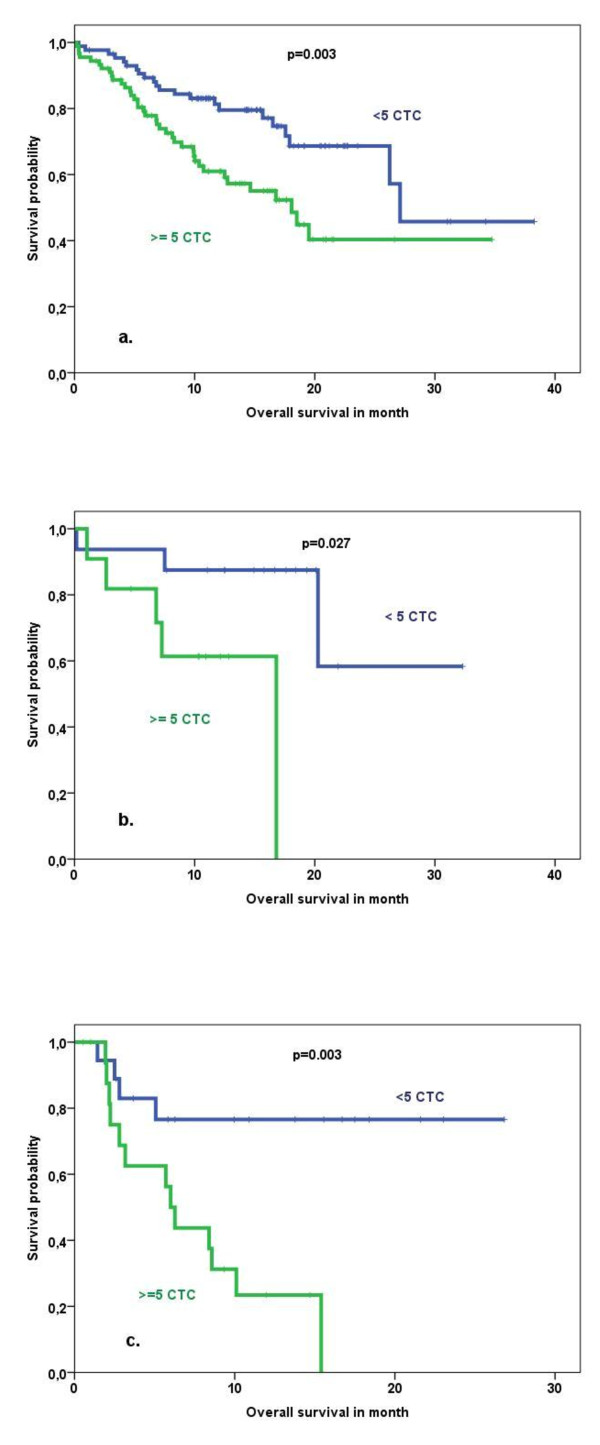
**Overall survival in correlation to circulating tumor cell (CTC) detection with the CellSearch™ system subdivided by type of the primary tumor**. **(a) **For 'luminal' type, for example, hormone receptor-positive and HER2-negative. **(b) **For HER2-positive. **(c) **For triple-negative primary tumors. HER2, human epidermal growth factor receptor 2.

With the established cutoff level of 5 or more CTCs, 122 out of 245 (50%) metastatic patients were considered CTC-positive. The PFS was not correlated with positive CTC status defined as 5 or more CTCs (Figure 1 and Table 1). The mean rates of OS were 18.1 months in CTC-positive patients (95% CI 15.1 to 22.1 months) and 27 months in CTC-negative patients (23.5 to 30.7 months; *P *< 0.001) (Table 1 and Figure 2). In multivariate analysis including CTC positivity, molecular subtype and number and sites of metastatic disease, the presence of CTCs was an independent predictor for OS (hazard ratio 2.7, 95% CI 1.6 to 4.2) (Table 2). The same analysis was performed with a cutoff of one CTC. The positivity rate was 73.5% (180 out of 245 patients). CTC positivity was also associated with reduced OS. However, the cutoff of 5 cells was associated with a higher risk of death (Table 2).

**Table 1:** Mean overall survival and progression-free survival in metastatic breast cancer subdivided on the basis of circulating tumor cell positivity

	OS, months	95% CI, months	*P *value	PFS, months	95% CI, months	*P *value
Total	23.174	20.4-25.9	-	10.2	9.1-11.3	-
CellSearch™						
≥1 CTC(s)	19.6	17.1-22.2	<0.01	9.8	8.5-11.0	n.s.
<1 CTC	30.1	26.3-33.9		10.9	8.8-12.9	
≥5 CTCs	18.1	15.1-21.1	<0.01	9.3	7.8-10.9	
<5 CTCs	27.1	23.5-30.7		10.9	9.3-12.5	n.s.
**AdnaTest Breast™**						
CTC-positive	19.3	15.4 - 23.1	n.s.	8.8	7.2-10.4	n.s.
CTC-negative	23.8	20.3-27.5		10.7	9.2-12.2	

CTC, circulating tumor cell; n.s., not significant; OS, overall survival; PFS, progression-free survival.

**Table 2:** Univariate and multivariate analyses of overall survival

	Univariate	Multivariate	
	*P *value	OR (95% CI)	*P *value
Menopausal status			
Postmenopausal Premenopausal	0.52	-	-
ER status			
Positive	0.26	-	-
Negative			
PR status			
Negative	0.47	-	-
Positive			
HER2 status			
Negative	0.30	-	-
Positive			
Metastatic site			
Bone	0.01	1	0.04
Visceral		2.6 (1.0-6.8)	0.05
Both		3.2 (1.3-8.2)	0.01
Number of metastatic sites			
One site Multiple sites	0.01	Not significant	-
Disease-free interval			
≤12 months	0.12	-	-
>12 months			
CTC positivity			
<5 cells	0.00	1	<0.01
≥5 cells		2.7 (1.6-4.2)^a^	
Subtype			
'Luminal'	0.09	1	0.02
HER2-positive		0.9 (0.4-2.0)	0.84
Triple-negative		2.1 (1.2-3.8)	0.00

^a^Based on cutoff level of one circulating tumor cell (CTC): 2.06 (1.16-3.68), *P *< 0.05. CI, confidence interval; ER, estrogen receptor; HER2, human epidermal growth factor receptor 2; OR, odds ratio; PR, progesterone receptor.

No relevant difference between CTC detection rates in patients with different subtypes of the primary tumor defined as HER2-positive versus hormone receptor-positive/HER2-negative ('luminal') versus triple-negative was observed (*P *= 0.619) (Table 2). Regardless of the molecular subtype, CTC positivity was associated with reduced OS (Figure 2 and Table 3) but not with PFS (data not shown).

**Table 3:** Prognostic significance of circulating tumor cells for overall survival subdivided by subtype of primary tumor

Subtype of primary tumor	Overall survival in months <5 CTCs	Overall survival in months ≥5 CTCs	*P *value
Luminal^a^	21.2 (16.5-26.5)	7.4 (4.8-10.1)	0.003
HER2 subtype	25.2 (18.3-32.1)	12.1 (7.9-16.2)	0.027
Triple-negative	26.7 (22.6-30.9)	19.7 (16.2-23.1)	0.003

Values of overall survival are presented as mean (95% confidence interval). ^a^Hormone receptor-positive and HER2-negative. CTC, circulating tumor cell; HER2, human epidermal growth factor receptor 2.

### Prognostic impact of the AdnaTest BreastCancer™

When the AdnaTest Breast was performed, 88 out of 221 (40%) patients were CTC-positive. Concordance between the two assays used in our study was 64% (*P *< 0.01, κ = 0.28) as described earlier [14]. No correlation could be observed between CTC positivity and any of the analyzed clinicopathological factors, except for HER2 status. CTC positivity assessed by the AdnaTest Breast™ had no association with clinical outcome parameters such as PFS or OS. Median PFS values were 8.8 and 10.7 months (*P *= 0.230), and OS values were 19.3 and 23.8 (*P *= 0.278). This was also the case when patients were analyzed according to the different subtypes of their primary tumors (data not shown). Therefore, a multivariate analysis was not performed.

## Discussion

In this study, we used the CellSearch™ system and AdnaTest BreastCancer™ because they are supposed to be suitable tools for CTC detection in a multicenter setting due to standardized procedures. With a reasonable follow-up period, we describe the prognostic impact of the two methods and find a prognostic value for the CellSearch™ system only.

The FDA-cleared CellSearch™ system is currently the most frequently used approach, particularly in ongoing clinical trials. CTCs are isolated by immunomagnetic beads coated with antibodies against EpCAM and identified by cytokeratin positivity, positive nuclear staining, and CD45 negativity. Using only EpCAM to enrich CTCs from blood is considered one of the major limitations of this assay [17]. EpCAM might be heterogeneously expressed by CTCs and be downregulated as a consequence of the metastatic process [18]. In addition, experimental data suggest that normal-like breast cancer cells are less efficiently captured by an EpCAM-based approach, resulting in a lower sensitivity of this assay. It has been speculated that patients with triple-negative primary tumors have a higher rate of normal-like CTCs [19]. However, we observed no relevant differences in detection rates or prognostic impact between patients with different subtypes of primary tumors, including those with triple-negative primary tumors. The prognostic impact in this subgroup seems to be strong (Figure 2 and Table 3). Therefore, CTC detection could help to identify patients who are candidates for additional targeted treatment also in clinical trials since treatment options in this group of patients are limited.

We observed a prognostic impact of CTC detection also in the subgroup of patients with HER2-positive tumors. Giordano and colleagues [20] found a prognostic impact of CTC detection in all subgroups, except for patients with HER2-positive tumors that received targeted therapy. This does not indicate a complete disagreement with our findings since in our cohort not all patients received anti-HER2 treatment in further lines of treatment.

The clinical potential to improve patient outcome with CTC detection is not yet completely clear. CTC detection could help to identify patients who need more aggressive therapy and to monitor treatment with repeated testing. To examine this potential, the US SWOG 0500 trial [21] and the French trial CirCe01 [22] were initiated but have not reported results. Our study group has started the DETECT III study to compare standard therapy alone versus standard therapy plus lapatinib in patients with initially HER2-negative metastatic breast cancer and HER2-positive CTCs [23]. In this context, our results support the use of the CellSearch™ system with the currently established cutoff of 5 CTCs in these and other trials on patients with metastatic breast cancer. We did not observe a better prognostic discrimination with the cutoff of one CTC (Table 1). However, in patients with less advanced disease (that is, patients with primary breast cancer), the prognostic significant cutoff value is one CTC, as shown in the SUCCESS (Simultaneous Study of Gemcitabine-Docetaxel Combination adjuvant treatment, as well as Extended Bisphosphonate and Surveillance-Trial) trial [24].

The lack of correlation between CTC detection and PFS in our cohort could have been caused mainly by different definitions of disease progression based on institutional standards of the participating centers. This could make the endpoint of PFS less reliable.

AdnaTest BreastCancer™ was the other CTC test used in our study. Here, CTCs are isolated by immunomagnetic beads labeled with antibodies against MUC1 and EpCAM. The potential advantage of this approach is the possibility to characterize cells simultaneously for several additional markers like HER2 and steroid receptors. In the present study, the overall detection rate for CTCs was 40%, which is within in the range of other published studies for metastatic breast cancer [25,26]. The fact that we did not observe a prognostic impact of CTC detection with this method is, in our view, hard to explain by any confounding factors of the cohort examined. Also, no prognostic impact was observed in different subgroups of primary tumors. A possible explanation is the choice of the marker in the AdnaTest: MUC1 can be also expressed by activated leukocytes, and the mRNA expression of this marker is, therefore, not restricted to CTCs. This may lead to false-positive findings in particular in patients undergoing cytotoxic therapies associated with massive cell death and induction of inflammation and other processes (such as autophagy) that affect the activation status of leukocytes.

Two other publications have compared the detection rates of CTCs between the CellSearch™ system and AdnaTest BreastCancer™ but did not provide clinical follow-up data. Andreopoulou and colleagues [27] examined 55 patients with metastatic breast cancer. The authors found positive rates of 53% for the AdnaTest and 36% for the CellSearch™ system with a cutoff of 5 CTCs. Van der Auwera and colleagues [25] examined 76 patients with metastatic breast cancer and found positive rates of 36% by the CellSearch™ system and 22% by the AdnaTest Breast. In our view, the observed differences concerning the positivity rate, especially for the AdnaTest, might be due to different percentages of HER2-positive CTCs in bitgh studies since CTC detection with the AdnaTest Breast is also based on HER2 as one of the transcripts used by the assay for CTC detection. Our detection rate with the AdnaTest Breast is 40%, which is between the results of the two publications. In our study, the AdnaTest was considered to be positive if a PCR fragment of at least one tumor-associated transcript was clearly detected with the cutoff of 0.15 ng/µL recommended by the manufacturer and used by Andreopoulou and colleagues [27]. In one other publication, the cutoff for positivity was considered inconclusive between 0.15 and 30 ng/µL [25]. This could also account for some of the differences between studies.

Our study is the largest one to compare CTC testing with different methods that provide a clinical follow-up. Advantages are the blinded data entry and technical performance of the assays in the multicenter setting. The lack of standardized treatment and response monitoring is a potential drawback of the study but reflects the clinical routine setting.

## Conclusions

We demonstrated, in a comparative and prospective multicenter study, that CTC detection had prognostic impact if the results were obtained with the CellSearch™ system but not with the AdnaTest Breast. This finding has important implications for the future of CTCs as novel biomarkers. Given the fact that many promising new assays for CTC detection are in development [28], further evaluation of these assays should also include their validation in a relevant clinical setting with sufficient follow-up information and OS as the objective endpoint.

## Abbreviations

bp: base pairs; CI: confidence interval; CTC: circulating tumor cell; EpCAM: epithelial cell adhesion molecule; ER: estrogen receptor; FDA: US Food and Drug Administration; HER2: human epidermal growth factor receptor 2; OS: overall survival; PCR: polymerase chain reaction; PFS: progression-free survival; PR: progesterone receptor; RT-PCR: reverse transcription-polymerase chain reaction.

## Competing interests

WJ and KP have received educational grants from Veridex, LLC. TF has received research support from Adnagen AG. The other authors declare that they have no competing interests.

## Authors' contributions

VM and TF drafted the paper. All authors participated in patient recruitment, conception of the DETECT study, and acquisition, analysis, and interpretation of data and revised the paper critically for important intellectual content. VM, SR,, TF and KP contributed equally to the manuscript. All authors read and approved the final manuscript.
